# Supplementary material for: Healthcare utilization for atopic dermatitis: An analysis of the 2010–2018 health insurance review and assessment service national patient sample data
Source: PLoS One. 2023 Jun 26;18(6):e0286449. doi: 10.1371/journal.pone.0286449 (PMC10292712; doi:10.1371/journal.pone.0286449)
Supplement: S1 Table — (DOCX) [file pone.0286449.s001.docx]

**Healthcare utilization for atopic dermatitis: An analysis of the 2010-2018 Health Insurance Review and Assessment Service National Patient Sample Data**

Sowon Kim^1†^, Ye-Seul Lee^2†^, Jiyoon Yeo^2^, Donghyo Lee^3^, Ko Dong Kun^4^, In-Hyuk Ha^2*^

^1^ Jaseng Hospital of Korean Medicine, Gangnam-daero, Gangnam-gu, Seoul, Republic of Korea

^2^ Jaseng Spine and Joint Research Institute, Jaseng Medical Foundation, Gangnam-daero, Gangnam-gu, Seoul, Republic of Korea

^3^ Department of Ophthalmology, Otolaryngology, and Dermatology, College of Korean Medicine, Woo-Suk University, Jeonju, Korea

^4^ Jayeonsaeng Korean Medicine Clinic, Yongin, Korea

† Both authors are co-first authors.

***Corresponding author:** In-Hyuk Ha

Jaseng Spine and Joint Research Institute

Jaseng Medical Foundation

3F, 538 Gangnam-daero

Gangnam-gu, Seoul 06110, Republic of Korea

E-mail: [hanihata@gmail.com](mailto:hanihata@gmail.com) (IHH)

**Table S1.** Annual average KRW-USD exchange rate and price level of health expense

| **Year** | **KRW/USD** | **Price level** |
| --- | --- | --- |
| 2010 | 1,156.00 | 0.9345 |
| 2011 | 1,107.99 | 0.9510 |
| 2012 | 1,126.76 | 0.9597 |
| 2013 | 1,095.04 | 0.9631 |
| 2014 | 1,053.12 | 0.9699 |
| 2015 | 1,131.52 | 0.9820 |
| 2016 | 1,160.41 | 0.9918 |
| 2017 | 1,130.48 | 1.0005 |
| **2018** | **1,100.58** | **1.0000** |
| This information is available from the following site: Korean Statistical Information Service (<http://kosis.kr>).  The price level of health expense is adjusted as of 2018. | | |

Supplementary Figure 4 9-Year trend of 원내처방 drug use to treat atopic dermatitis
