# Supplementary material for: Healthcare utilization for atopic dermatitis: An analysis of the 2010–2018 health insurance review and assessment service national patient sample data
Source: PLoS One. 2023 Jun 26;18(6):e0286449. doi: 10.1371/journal.pone.0286449 (PMC10292712; doi:10.1371/journal.pone.0286449)
Supplement: S2 Table — (DOCX) [file pone.0286449.s002.docx]

**Healthcare utilization for atopic dermatitis: An analysis of the 2010-2018 Health Insurance Review and Assessment Service National Patient Sample Data**

Sowon Kim^1†^, Ye-Seul Lee^2†^, Jiyoon Yeo^2^, Donghyo Lee^3^, Ko Dong Kun^4^, In-Hyuk Ha^2*^

^1^ Jaseng Hospital of Korean Medicine, Gangnam-daero, Gangnam-gu, Seoul, Republic of Korea

^2^ Jaseng Spine and Joint Research Institute, Jaseng Medical Foundation, Gangnam-daero, Gangnam-gu, Seoul, Republic of Korea

^3^ Department of Ophthalmology, Otolaryngology, and Dermatology, College of Korean Medicine, Woo-Suk University, Jeonju, Korea

^4^ Jayeonsaeng Korean Medicine Clinic, Yongin, Korea

† Both authors are co-first authors.

***Corresponding author:** In-Hyuk Ha

Jaseng Spine and Joint Research Institute

Jaseng Medical Foundation

3F, 538 Gangnam-daero

Gangnam-gu, Seoul 06110, Republic of Korea

E-mail: [hanihata@gmail.com](mailto:hanihata@gmail.com) (IHH)**Table S2.** **Basic characteristics of Korean and Western medicine usage**

| **Category** | | **Total (2010-2018)** | | **WM (2010-2018)** | | **KM (2010-2018)** | |
| --- | --- | --- | --- | --- | --- | --- | --- |
| **Type of visit** | Outpatient | 631,850 | 99.94 | 556,839 | 99.94 | 75,011 | 99.94 |
|  | Inpatient | 408 | 0.06 | 362 | 0.06 | 46 | 0.06 |
| **Medical institution** | Tertiary hospital/general hospital/hospital | 75,362 | 11.92 | 75,282 | 13.51 | 80 | 0.11 |
|  | Clinic | 481,855 | 76.21 | 481,855 | 86.48 | - | - |
|  | KM hospital | 1,931 | 0.31 | 64 | 0.01 | 1,867 | 2.49 |
|  | KM clinic | 73,110 | 11.56 | - | - | 73,110 | 97.41 |

WM, Western medicine; KM, Korean medicine

All expenses are converted to USD from KRW according to the annual average exchange rate (see Table S1).
