# Supplementary material for: Healthcare utilization for atopic dermatitis: An analysis of the 2010–2018 health insurance review and assessment service national patient sample data
Source: PLoS One. 2023 Jun 26;18(6):e0286449. doi: 10.1371/journal.pone.0286449 (PMC10292712; doi:10.1371/journal.pone.0286449)
Supplement: S3 Table — (DOCX) [file pone.0286449.s003.docx]

**Healthcare utilization for atopic dermatitis: An analysis of the 2010-2018 Health Insurance Review and Assessment Service National Patient Sample Data**

Sowon Kim^1†^, Ye-Seul Lee^2†^, Jiyoon Yeo^2^, Donghyo Lee^3^, Ko Dong Kun^4^, In-Hyuk Ha^2*^

^1^ Jaseng Hospital of Korean Medicine, Gangnam-daero, Gangnam-gu, Seoul, Republic of Korea

^2^ Jaseng Spine and Joint Research Institute, Jaseng Medical Foundation, Gangnam-daero, Gangnam-gu, Seoul, Republic of Korea

^3^ Department of Ophthalmology, Otolaryngology, and Dermatology, College of Korean Medicine, Woo-Suk University, Jeonju, Korea

^4^ Jayeonsaeng Korean Medicine Clinic, Yongin, Korea

† Both authors are co-first authors.

***Corresponding author:** In-Hyuk Ha

Jaseng Spine and Joint Research Institute

Jaseng Medical Foundation

3F, 538 Gangnam-daero

Gangnam-gu, Seoul 06110, Republic of Korea

E-mail: [hanihata@gmail.com](mailto:hanihata@gmail.com) (IHH)

**Table S3.** **Top 10 WM/KM sub-diagnoses**

| **WM** | | | **Frequency** | **Percentage** | **Cumulative frequency** | **Cumulative percentage** |
| --- | --- | --- | --- | --- | --- | --- |
| **Rank** | **Diagnosis** | |  |  |  |  |
| 1 | L23.9 | Allergic contact dermatitis, unspecified cause | 16,625 | 8.92 | 16,625 | 8.92 |
| 2 | J30.4 | Allergic rhinitis, unspecified | 10,171 | 5.46 | 26,796 | 14.37 |
| 3 | L50.9 | Urticaria, unspecified | 6,818 | 3.66 | 33,614 | 18.03 |
| 4 | K29.7 | Gastritis, unspecified | 6,587 | 3.53 | 40,201 | 21.57 |
| 5 | L29.9 | Pruritus, unspecified | 6,178 | 3.31 | 46,379 | 24.88 |
| 6 | L50.0 | Allergic urticaria | 6,118 | 3.28 | 52,497 | 28.16 |
| 7 | L23.8 | Allergic contact dermatitis due to other agents | 5,950 | 3.19 | 58,447 | 31.35 |
| 8 | K30 | Functional dyspepsia | 5,361 | 2.88 | 63,808 | 34.23 |
| 9 | L01.0 | Impetigo [any organism][any site] | 4,452 | 2.39 | 68,260 | 36.62 |
| 10 | J20.9 | Acute bronchitis, unspecified | 4373 | 2.35 | 72,633 | 38.96 |
|  |  | | | | | |
| **KM** | | | **Frequency** | **Percentage** | **Cumulative frequency** | **Cumulative percentage** |
| **Rank** | **Diagnosis** | |  |  |  |  |
| 1 | J00 | Acute nasopharyngitis[common cold] | 396 | 16.22 | 396 | 16.22 |
| 2 | K30 | Functional dyspepsia | 283 | 11.59 | 679 | 27.81 |
| 3 | J30.4 | Allergic rhinitis, unspecified | 182 | 7.45 | 861 | 35.26 |
| 4 | J31.0 | Chronic rhinitis | 111 | 4.55 | 972 | 39.80 |
| 5 | M62.6 | Muscle strain | 86 | 3.52 | 1,058 | 43.33 |
| 6 | M79.1 | Myalgia | 61 | 2.50 | 1,119 | 45.82 |
| 7 | R10.4 | Other and unspecified abdominal pain | 59 | 2.42 | 1,178 | 48.24 |
| 8 | M54.5 | Low back pain | 51 | 2.09 | 1,229 | 50.33 |
| 9 | J30.3 | Other allergic rhinitis | 50 | 2.05 | 1,279 | 52.38 |
| 10 | K59.0 | Constipation | 42 | 1.72 | 1,321 | 54.10 |
| ICD-10 Version: Based on 2019 version  WM, Western medicine; KM, Korean medicine | | | | | | |
