# Supplementary material for: Healthcare utilization for atopic dermatitis: An analysis of the 2010–2018 health insurance review and assessment service national patient sample data
Source: PLoS One. 2023 Jun 26;18(6):e0286449. doi: 10.1371/journal.pone.0286449 (PMC10292712; doi:10.1371/journal.pone.0286449)
Supplement: S4 Table — (DOCX) [file pone.0286449.s004.docx]

**Healthcare utilization for atopic dermatitis: An analysis of the 2010-2018 Health Insurance Review and Assessment Service National Patient Sample Data**

Sowon Kim^1†^, Ye-Seul Lee^2†^, Jiyoon Yeo^2^, Donghyo Lee^3^, Ko Dong Kun^4^, In-Hyuk Ha^2*^

^1^ Jaseng Hospital of Korean Medicine, Gangnam-daero, Gangnam-gu, Seoul, Republic of Korea

^2^ Jaseng Spine and Joint Research Institute, Jaseng Medical Foundation, Gangnam-daero, Gangnam-gu, Seoul, Republic of Korea

^3^ Department of Ophthalmology, Otolaryngology, and Dermatology, College of Korean Medicine, Woo-Suk University, Jeonju, Korea

^4^ Jayeonsaeng Korean Medicine Clinic, Yongin, Korea

† Both authors are co-first authors.

***Corresponding author:** In-Hyuk Ha

Jaseng Spine and Joint Research Institute

Jaseng Medical Foundation

3F, 538 Gangnam-daero

Gangnam-gu, Seoul 06110, Republic of Korea

E-mail: [hanihata@gmail.com](mailto:hanihata@gmail.com) (IHH)

**Table S4.** **Inpatient prescription**

| **Full name** | **Total Claims** | **Percentage (out of total claims)** | **Average cost per claim** | **Average cost per patient** |
| --- | --- | --- | --- | --- |
| Betamethasone | 20,147 | 3.19% | 0.32 | 0.72 |
| Dexamethasone | 32,996 | 5.22% | 0.13 | 0.27 |
| Triamcinolone | 4,748 | 0.75% | 0.40 | 0.79 |
| Hydroxine | 81,306 | 12.86% | 0.06 | 0.12 |
| Diphenylpyraline | 10,009 | 1.58% | 0.22 | 0.52 |
| Chlorphenamine | 57,566 | 9.10% | 0.06 | 0.13 |

All expenses are converted to USD from KRW according to the annual average exchange rate (see Table S1).

Supplementary Figure 4 9-Year trend of 원내처방 drug use to treat atopic dermatitis
