# Supplementary material for: Healthcare utilization for atopic dermatitis: An analysis of the 2010–2018 health insurance review and assessment service national patient sample data
Source: PLoS One. 2023 Jun 26;18(6):e0286449. doi: 10.1371/journal.pone.0286449 (PMC10292712; doi:10.1371/journal.pone.0286449)
Supplement: Attachment — Submitted filename: Answer to reviewers comments_230126.docx [file pone.0286449.s005.docx]

**Additional Editor Comments:**

**Thank you for submitting your manuscript. The reviewers and I believe it is of potential value for our readers. However, the reviewers have raised a number of very important issues, and their excellent comments will need to be adequately addressed in a revision before the acceptability of your manuscript for publication in the Journal can be determined. We cannot guarantee that your revised paper will be chosen for publication; this would be solely based on how satisfactorily you have addressed the reviewer comments.**

- We would like to thank the Editor for giving us a chance to revise the manuscript. We believe we’ve addressed all of the reviewer’s comments and, thanks to the thoughtful comments, the overall quality of the manuscript has improved.

**Reviewers' comments:**

**Reviewer #1: Overall it is interesting to compare the use of Western vs Korean Medicine in such a large cohort. Be careful with making broad claims about effectiveness or adverse effects in the introduction and discussion. The potential harms of CAM needs to be addressed. Could be considered for review if some of these issues are addressed and suggest specific changes as below:**

**Some of the language used could be improved and rewritten. Some examples:**

**In the abstract:**

**Line 28-29:**

**This sentence is vague and could be improved by including specific treatments. Which intramuscular, subcut and IV injections?:**

**The most used Western medicine treatments included intramuscular, subcutaneous, and intravenous injections, while it was acupuncture therapy in Korean medicine.**

- We appreciate the reviewer’s thoughtful comment. We revised the manuscript as follows:

- The most used Western medicine treatments were injections and oral medications involving topical corticosteroids, antihistamine agents, and oral steroids, while it was acupuncture therapy in Korean medicine. The frequency of the most frequently prescribed medication, topical corticosteroid, showed a decreasing trend over time.

**Line 30:**

**Perhaps use the word prescription medication rather than drug:**

**The most used drug was topical corticosteroid, but its frequency of use also showed a decreasing trend.**

- We appreciate the reviewer’s thoughtful comment. We revised the manuscript as follows:

- The frequency of the most frequently prescribed medication, topical corticosteroid, showed a decreasing trend over time.

**Line 30-32:**

**Could you expand on the relevance of the study ie. How many countries use acupuncture to treat atopic dermatitis so this study may only be of particular relevance to those countries.**

**The healthcare usage and trends in Western medicine and Korean medicine treatment for atopic dermatitis in this study provide baseline data for health policy makers and clinicians.**

- We appreciate the reviewer’s thoughtful comment. We revised the manuscript in the Abstract and in Introduction as follows:

**Abstract**

The findings in this study will inform healthcare policy makers and clinicians across different countries on the usage trends of Western medicine and Korean medicine treatment.

**Introduction**

Furthermore, a recent review found that the effectiveness of acupuncture in atopic dermatitis is being explored in other countries including China and Germany.

**Line 35-36:**

**“Atopic dermatitis (AD) commonly occurs in childhood and is characterized by chronically relapsing rashes and itching”**

**Could be reworded to something like:**

**Atopic Dermatitis (AD) is an inflammatory chronic and relapsing condition that presents with erythematous, scaly, pruritic rashes. It is most common in childhood, however all age groups can be affected.**

- We appreciate the reviewer’s thoughtful comment. We revised the manuscript as follows:

- Atopic dermatitis (AD) is a chronic and relapsing inflammatory condition that presents with erythematous, scaly, itchy rashes. It is most prevalent in children, but all age groups can be affected [1, 2].

**Line 36-37**

**I’m not sure that recent evidence has shown that it is occurs in older age groups, I believe older age groups have been known for some time.**

- We appreciate the reviewer’s thoughtful comment. We revised the manuscript as follows:

- Recent evidence indicates that it causes changes in the skin barrier and impairs immune function, increasing vulnerability to this disease throughout lifetime [3, 4].

**Line 53:**

**I’m not sure what you mean by AD can cause localized eczema?**

- We appreciate the reviewer’s thoughtful comment. We revised the manuscript as follows:

- From a personal health perspective, it can also have a negative impact by causing chronic skin diseases such as urticaria and alopecia; sleep disturbance due to itching; as well as complications of comorbid asthma and food allergies [18].

**Line 60:**

**“However, an optimal treatment strategy that is individualized according to disease severity, has not been established.” - ?in korea, there are multiple clinical practice guidelines in other countries**

- We appreciate the reviewer’s thoughtful comment. We revised the manuscript as follows:

- However, a long-term therapy plan that avoids corticosteroid adverse effects while enhancing their efficacy has always been the subject of controversy. Although more aggressive therapies involving systemic immunosuppressive medications, e.g., cyclosporine and mycophenolate, phototherapy, and allergen-immunotherapy is recommended for severe AD, an actual application and compliance in clinical settings have been reported to be relatively low.

**When discussing CAM – you need to address the risks and side effects of CAM such as side effects from herbal medicines including systemic toxicities, allergic contact dermatitis, cost, risk of avoiding Western or conventional medicine and hence risk of leaving disease untreated, worsening of disease and risk of complications such as infection or scarring.**

- We appreciate the reviewer’s thoughtful comment. We revised the manuscript as follows:

- Furthermore, potential risk of inappropriate use of CAM which may induce allergic reactions, additional burden of cost, and possible negligence of conventional medication by patients which may accelerate disease progress needs to be taken into account when analyzing utilization of CAM.

**The different insurance types need to be explained for the non-Korean audience.**

- We appreciate the reviewer’s thoughtful comment. We revised the manuscript as follows:

- The single insurer system in Korea by National Health Insurance (NHI), which supports 98% of all population and also provides data for 2% of the population who receive Medical Aid, provides healthcare coverage in both Western and Korean Medicine due to its unique dual healthcare system. Therefore, examining the NHI database provides knowledge on healthcare spendings from societal perspective in both Western and Korean Medicine.

**Line 219: CBC ?complete blood count, is this written in full earlier, otherwise be careful of abbreviations.**

- We appreciate the reviewer’s thoughtful comment. We revised the manuscript as follows:

- The second and third highest average per-patient expense was for other tests ($83.42) and complete blood count (CBC) test (Code B1~: $29.22), respectively, indicating that testing fees accounted for a large portion of the cost burden for patients.

**Line 232-233:**

**“This was followed by Gamma-linolenic acid, Staphylococcus aureus, and Pityrosporum ovale.”**

**I don’t think this sentence makes sense. It indicates staph aureus is a treatment when it is a bacterium?**

- We appreciate the reviewer’s thoughtful comment. We revised the manuscript as follows:

- . This was followed by antibiotics for topical use & for systemic use, antifungals for topical use & antimycotics for systemic use, and gamma-linolenic acid. The highest average expense per case was for antifungals for topical use & antimycotics for systemic use, while the highest average per-patient expense was for gamma-linolenic acid.

**Line 235-236:**

**What does optimal drug treatments mean?**

- We appreciate the reviewer’s thoughtful comment. We revised the manuscript and changed the term “drug treatments” to prescribed medications or prescription medication. Examples of the changes are as follows:

- Among optional prescription medications, oral steroids showed the highest number of claims at 163,811, accounting for approximately 47% of all optional prescriptions.

**Line 310-311**

**Herbal concoctions, such as Oryeong-san [39], GagamBangpungtongseong-san [40], and Gammakdaejo311tang [41] are effective in treating AD.**

**This is a broad sweeping claim. I don’t think that one case study and two animal studies can demonstrate effectiveness.**

- We appreciate the reviewer’s thoughtful comment. We revised the manuscript as follows:

**-** Herbal concoctions, such as Oryeong-san [41], GagamBangpungtongseong-san [42], and Gammakdaejo-tang [43] have been presented as potential options for AD, although further clinical studies are required to confirm the effectiveness.

**Line 320-321:**

**Systemic toxicities such as cushings syndrome are rare, and linked to inappropriate and overuse and this needs to be discussed.**

**I don’t believe there is a risk of lymphoma and TCS use, https://pubmed.ncbi.nlm.nih.gov/25840730/**

- We appreciate the reviewer’s thoughtful comment. We revised the manuscript as follows:

- The decreased usage of TCS could be a result of preventive measures against skin AEs like skin atrophy and telangiectasis; further, systemic AEs like hypothalamic-pituitary-adrenal inhibition and Cushing’s syndrome have also been suggested, although it’s extremely rare and often linked to overuse [50, 51].

**Line 322:**

**Reframe this sentence to reference what this study showed. Saying that TCS increase psychological problems is a broad sweeping statement and may not apply to all cohorts. Otherwise this needs to be demonstrated with more evidence and references:**

**“compared with no treatment, topical and systemic steroid treatment is associated with a three times higher risk of psychological problems [50].”**

- We appreciate the reviewer’s thoughtful comment. Upon second assessment of our manuscript, we decided that this sentence and reference does not adequately support the context in the paragraph and therefore removed them.

**340-341:**

**Might be worth mentioning upadacitinib here as a treatment option too.**

- We appreciate the reviewer’s thoughtful comment. We revised the manuscript as follows:

- Recent findings indicate upadacitinib as an option for moderate to severe atopic dermatitis[56]; however, it has been covered by NHI since May of 2022 in Korea, therefore it was not included in this study.

**Reviewer #2: I have no further comments regarding this, this is a standard, good paper, not overly exciting, however it will possibly find it's purpose in everyday practice. Although it is to an extent common wisdom, it is well organized, has a point and shows in a systematic manner the idea how to improve access to health care utilization in AD patients**

- We thank the reviewer for the thoughtful comment.

**Reviewer #3: Dear authors,**

**I have now completed the review of the manuscript titled "Healthcare utilization for atopic dermatitis: an analysis of the 2010–2018 Health Insurance Review and Assessment Service National Patient Sample Data."**

**In the present study, the authors analyzed the distribution and healthcare usage patterns of patients with atopic dermatitis using the 2010-2018 Health Insurance Review and Assessment Service data.**

**The manuscript is interesting and, in general, fair written.**

**I would like to recommend an "accept" decision, but before doing so, I would like to suggset one minor suggestion.**

**In the ‘Statistical analysis’ section, there are no citing references, therefore I would like to suggest adding the following reference in the sentence:**

**The average annual log change for each item, and the differences between WM and KM, were investigated[1, 2].**

[1] https://doi.org/10.54724/lc.2022.e1

[2] https://doi.org/10.54724/lc.2022.e3

**I hope good luck to the authors' future work and research. Thank you.**

- We appreciate the reviewer’s thoughtful comment. We revised the manuscript as follows:

The average annual log change for each item, and the differences between WM and KM, were investigated[30, 31].
