# Supplementary material for: Healthcare utilization for atopic dermatitis: An analysis of the 2010–2018 health insurance review and assessment service national patient sample data
Source: PLoS One. 2023 Jun 26;18(6):e0286449. doi: 10.1371/journal.pone.0286449 (PMC10292712; doi:10.1371/journal.pone.0286449)
Supplement: Attachment — Submitted filename: Review Comments to the Author_230426.docx [file pone.0286449.s006.docx]

Review Comments to the Author

**Reviewer #1: While the authors revised some of their sections, they didn’t adequately address concerns that I had during my first review.**

**Issues that need to be addressed: biased and generalized interpretation of results in discussion, not adequately contextualized the relevance of their study in the broader dermatology community.**

- We appreciate the reviewer’s comment. Based on the reviewer’s comment, we thoroughly revised the Introduction and paragraphs in the Discussion where CAM therapies were being discussed. Some of the examples are found below:

With respect to healthcare usage, the number of patients who used WM was much higher than the number of patients who used KM. Among the WM patients, most were from primary medical institutions (clinics, 86.48%; tertiary/general/regular hospitals, 13.51%). The majority of healthcare users were in the age of <5 years (31.40%), 5–14 years (23.53%), and 15–24 years (15.33%), which may be attributed to the overall high prevalence of AD in the above age groups. There was no significant difference in healthcare usage by sex. The utilization trends by age groups and sex were similar across the types of medicine (WM, KM and WM+KM). The types of medical institutions, however, varied across the types of medicine; in specific, most KM utilization has taken place mostly in primary care, while more than 13% of WM utilization has taken place in tertiary care. For inpatient and outpatient care, the majority were outpatient cases (99.9% vs 0.06%).

**The study may have value in it’s objective results, however the write up fails to acknowledge existing literature and evidence in regards to CAM and other systemic treatments.**

- We appreciate the reviewer’s comment. Based on the reviewer’s comment, we thoroughly revised the sentences as follows:

Acupuncture was reported to improve AD symptoms, markedly reducing type 1 hypersensitivity itch [29, 43-46]; one previous study reported that cupping therapy is effective in reducing itchiness and lichenification in AD [47], although further study is warranted. Several herbal concoctions have been presented as potential options for AD [48-50], although further clinical studies are required to confirm the effectiveness. In this study, CAM therapies such as herbal decoctions were not included in the database since HIRA-NPS data only include medical services covered by national health insurance.

**One sentence on the damages of CAM (incorrectly worded as “inappropriate use of CAM”), does not adequately address this issue. There is significant and misinformed discussion about the side effects of conventional medicine which makes this paper appear biased and is promoting misinformation.**

- We appreciate the reviewer’s comment. Based on the reviewer’s comment, we thoroughly revised the sentences as follows and referenced the evidence with further accuracy:

While the effectiveness of CAM on AD has not been confirmed[26, 27], a few clinical trials and reviews have reported possible efficacy of acupuncture for symptom improvement and reduced recurrence across different populations [28-33], and one study reported a trend towards reduced TCS use in acupuncture and osteopathic treatment groups[34]. Nonetheless, it is crucial for both physicians and patients to note the uncertainties involved in CAM therapies due to lack of evidence on its safety and efficacy in AD management. The potential risk of inappropriate use of CAM which may induce allergic reactions, additional burden of cost, and possible negligence of conventional medication by patients which may accelerate disease progress needs to be taken into account when analyzing utilization of CAM.

**Line 60-63, this sentence is generalized and factually incorrect. This type of language, bias and poor representation of other treatment options is concerning. If there are studies that have found this, only one survey based study was referenced.**

- We appreciate the reviewer’s comment. Based on the reviewer’s comment, we thoroughly revised the sentences as follows:

Systemic immunosuppressants e.g., cyclosporine, methotrexate, and mycophenolate mofetil are recommended for severe AD, while caution is warranted to avoid side effects when long-term treatments are administered [19, 20]. One survey conducted to Korean dermatologists stated that systemic immunosuppressants are not actively used in clinical settings[21].

**Some of the references cited as strong evidence eg reference 20, 24, 25 are survey based or low evidence studies. This is not adequately acknowledged in the write up.**

- We appreciate the reviewer’s comment. Based on the reviewer’s comment, we thoroughly revised the sentences as follows:

While the effectiveness of CAM on AD has not been confirmed[26, 27], a few clinical trials and reviews have reported possible efficacy of acupuncture for symptom improvement and reduced recurrence across different populations [28-33], and one study reported a trend towards reduced TCS use in acupuncture and osteopathic treatment groups[34]. Nonetheless, it is crucial for both physicians and patients to note the uncertainties involved in CAM therapies due to lack of evidence on its safety and efficacy in AD management. The potential risk of inappropriate use of CAM which may induce allergic reactions, additional burden of cost, and possible negligence of conventional medication by patients which may accelerate disease progress needs to be taken into account when analyzing utilization of CAM.

**Reference 48:
The conclusion of the study of reference 48 states “However, in the adjusted analysis, severity of AD was the main factor associated with an increased risk of lymphoma.”
The authors use this reference to support their statement that topical corticosteroids cause lymphoma. This is concerned that the evidence is misconstrued and promotes hysteria and misinformation about topical corticosteroids.**

- We appreciate the reviewer’s comment. Based on the reviewer’s comment, we thoroughly revised the sentences and deleted the phrase that the reviewer pointed out. The paraphrased sentence is as follows:

The decreased usage of TCS could be the result of preventive measures against skin AEs like skin atrophy and telangiectasis, and in extremely rare cases systemic responses in the immune system [57, 58].
